# Enhancing Melanoma Diagnosis in Histopathology with Deep Learning and Synthetic Data Augmentation

**DOI:** 10.3390/bioengineering12091001

**Published:** 2025-09-21

**Authors:** Alex Rodriguez Alonso, Ana Sanchez Diez, Goikoane Cancho Galán, Rafael Ibarrola Altuna, Gonzalo Irigoyen Miró, Cristina Penas Lago, Mª Dolores Boyano López, Rosa Izu Belloso

**Affiliations:** 1 Department of Cell Biology and Histology, Faculty of Medicine and Dentistry, Biobizkaia Health Research Institute, University of the Basque Country, 48940 Leioa, Spain; alecai.rodriguez@gmail.com (A.R.A.); cristina.penas@ehu.eus (C.P.L.); lola.boyano@ehu.eus (M.D.B.L.); 2 Dermatology Department, Biobizkaia Health Research Institute, University Basurto Hospital, Avda. Montevideo 18, 48013 Bilbao, Spain; ana.sanchezdiez@osakidetza.eus; 3 Pathology Department, Biobizkaia Health Research Institute, University Basurto Hospital, Avda. Montevideo 18, 48013 Bilbao, Spain; goikoane.canchogalan@osakidetza.eus; 4 Pathology Department, Biobizkaia Health Research Institute, University Galdakao Hospital, Labeaga Auzoa, 48960 Galdakao, Bizkaia, Spain; rafael.ibarrolaaltuna@osakidetza.eus (R.I.A.); gonzalo.irigoyenmiro@osakidetza.eus (G.I.M.)

**Keywords:** melanoma, digital histopathology, synthetic images, GAN, automatic classification

## Abstract

Accurate diagnosis of melanoma using hematoxylin and eosin (H&E)-stained histological images is often challenged by the scarcity and imbalance of biomedical datasets, limiting the performance of deep learning models. This study investigates the impact of synthetic image generation, via generative adversarial networks (GAN), on training automatic classifiers based on the ResNet-18 architecture. Two experimental setups were designed: one using only real images and another combining real images with synthetic ones of the melanocytic nevus class to balance the dataset. Models were trained and evaluated at resolutions up to 1024 × 1024 pixels, employing standard classification metrics and the Fréchet Inception Distance (FID) to assess the quality of the generated images. The results suggest that although mixed models do not consistently outperform those trained exclusively on real data, they achieve competitive performance, particularly in terms of specificity and reduction in false negatives. This study supports the use of synthetic data as a complementary tool in scenarios where the acquisition of new samples is limited and lays the groundwork for future research in conditional generation and synthesis of malignant samples. In our best-performing model (1024 × 1024 px, 50 epochs, mixed dataset), we achieved an accuracy of 96.00%, a specificity of 97.00%, and a reduction in false negatives from 80 to 75 cases compared with real-only training. These results highlight the potential of synthetic augmentation to improve clinically relevant outcomes, particularly in reducing missed melanoma diagnoses.

## 1. Introduction

### 1.1. Melanoma Context and Diagnosis

Melanoma is one of the most aggressive forms of skin cancer, originating in melanocytes, the cells responsible for synthesizing melanin. Although it accounts for a relatively small percentage of all skin cancers, it is responsible for the majority of deaths related to these diseases due to its high metastatic potential and resistance to conventional therapies [1]. According to the Global Cancer Observatory, in 2020 more than 325,000 new cases of melanoma were diagnosed worldwide, with over 57,000 associated deaths [2].

Early diagnosis is a critical factor in patient prognosis. When melanoma is detected in its early stages, five-year survival rates exceed 90%; however, these rates drop drastically in advanced stages [3]. Traditionally, diagnosis is performed through clinical and dermatoscopic evaluation, followed by histopathological confirmation after biopsy. Histopathology, particularly using standard stains such as H&E, remains the gold standard for the classification of skin lesions [4].

Nevertheless, histopathological assessment presents significant limitations, such as inter-observer variability even among expert dermatopathologists and an increasing workload in pathology laboratories [5]. This context has driven growing interest in computer-aided diagnosis (CAD) methods, especially those based on deep learning.

### 1.2. Digital Histopathology and Machine Learning

The digitization of histological samples using high-resolution scanners has given rise to the field known as digital pathology, which enables the storage, visualization, and analysis of tissue sections through images known as Whole Slide Images (WSI). This evolution has created new opportunities for the development of computer-aided diagnostic tools by facilitating systematic access to large volumes of histopathological data [6].

**Figure 1:**
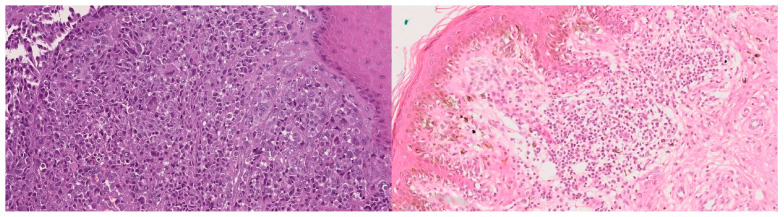
Histological comparison between malignant melanoma (**left**) and melanocytic nevus (**right**), stained with H&E.

In this context, deep learning has proven especially promising. Models such as Convolutional Neural Networks (CNN) have reached performance levels comparable to those of human experts in tasks such as tumor classification, metastasis detection, and cellular structure segmentation [7,8]. In the case of melanoma, several studies have shown that it is possible to train classification models with H&E-stained images, achieving clinically relevant results, particularly when distinguishing malignant melanomas from melanocytic nevi (Figure 1)—the main diagnostic challenge in pigmented lesions [9].

However, the performance of these models depends critically on the quantity and quality of the data available. In biomedical contexts, the number of labeled images is often limited, as annotation requires expert input. Moreover, datasets tend to be imbalanced, reflecting the low number of certain classes (such as melanocytic nevi) compared to others that are more frequent (such as invasive melanomas because these lesions are almost always biopsied). These conditions can induce overfitting and limit the generalization ability of trained models [10].

### 1.3. Limitations of Biomedical Data

Developing deep learning models in the biomedical domain faces structural limitations inherent to the available data. Unlike other domains such as computer vision for everyday objects, where massive datasets like ImageNet exist, in the clinical environment obtaining large volumes of labeled images is much more challenging.

First, biomedical image annotation requires specialist intervention (e.g., pathologists or dermatologists), which involves high time and resource costs. In addition, clinical data are subject to ethical, legal, and privacy restrictions, which limit their accessibility and sharing across institutions [11].

Second, these datasets are often imbalanced, reflecting the natural prevalence of diseases. In the case of melanoma, malignant samples are often much more frequent than melanocytic nevi, creating a bias that may impair model training [12]. This imbalance can lead to models with apparently high performance that fail to generalize well to underrepresented cases, which are often the most clinically relevant.

Another common challenge is technical variability: differences in acquisition conditions, scanner type, staining protocols, or image processing can introduce non-biological noise that affects model performance. This is known as the *batch effect* problem and can hinder model reproducibility in settings different from those in which it was originally trained [13].

To address these limitations, the use of artificially generated synthetic data has been proposed as a complementary solution to increase dataset diversity, balance, and size, thereby improving the robustness and generalization of trained models.

### 1.4. Motivation for Using Synthetic Images

The scarcity, imbalance, and variability of biomedical datasets have driven the exploration of strategies to artificially expand training sets without the need to acquire new clinical data. One of the most promising approaches in this field is the use of synthetic images generated through deep learning models, particularly GAN [14].

**Figure 2:**
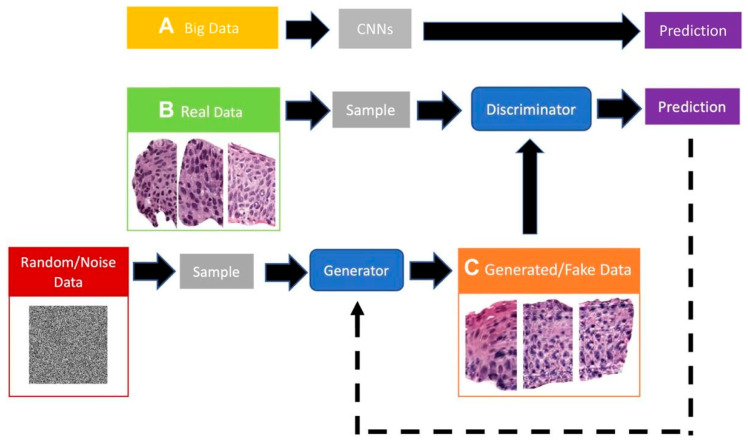
Schematic representation of a GAN architecture. Adapted from Alajaji et al. [16].

GANs allow the learning of the implicit probability distribution of a real dataset to generate new samples that, both visually and statistically, resemble the originals. This is especially valuable in histology, where images exhibit complex yet repetitive textural and structural patterns that can be effectively modeled by deep generative networks [15,16]. To improve accessibility for non-expert readers (e.g., clinicians), we retain a compact schematic of a GAN to visually anchor the concept (Figure 2).

The use of synthetic images offers several potential advantages:

- Increase the diversity of the training set, reducing the risk of overfitting.
- Balance minority classes by generating more examples of rare lesions, such as invasive melanomas.
- Simulate varied clinical conditions, including variability in staining, cell shape, and cell density, thereby improving classifier robustness.
- Protect patient privacy, as generated data cannot be directly linked to actual individuals.

Recent studies [17,18] have shown that incorporating synthetic images into classifier training can significantly improve performance, particularly in medical classification tasks with limited real data. However, the quality of the generated images and the method of integration into the training workflow are critical factors that must be carefully assessed. Conventional augmentation techniques such as rotations, flips, and color jittering can partially mitigate class imbalance and variability, but they do not generate new morphological structures. GAN-based synthesis offers the possibility of creating images with novel textural and architectural features, potentially providing a complementary benefit beyond traditional methods.

This work is grounded in this motivation, aiming to explore the impact of GAN-generated synthetic images on the performance of melanoma classifiers trained on H&E-stained histological images.

### 1.5. General Purpose of the Project

The main goal of this work is to evaluate the impact of using synthetic images—generated through GAN—on the performance of automatic melanoma classifiers trained on H&E-stained histological images.

This objective fits within the broader need to address inherent limitations of biomedical datasets—such as sample scarcity, class imbalance, and technical variability—by artificially generating data that complement and enhance real datasets.

This work received a favorable opinion from the Ethics Committee of Euskadi, under approval code PI+CES-BIOEF 2025-01, as recorded in Act No. 03/2025. This approval ensures that the use of anonymized histological data and the methodology applied comply with current ethical standards in biomedical research.

### 1.6. Hypothesis and Objectives

#### 1.6.1. Main Hypothesis

The hypothesis guiding this work is that the use of synthetic images generated through GAN contributes to improving the performance of melanoma classifiers trained on H&E-stained histological images. Specifically, it is expected that:

- The incorporation of synthetic images will increase the variability and size of the training set, thereby reducing overfitting.
- The class balance achievable through synthetic generation will improve the sensitivity and specificity of the model, particularly for minority classes.

This hypothesis is based on previous studies in which artificially generated data have proven effective in boosting the performance of models in clinical settings with limited data availability [16,17,18].

#### 1.6.2. Specific Objectives

To test the above hypothesis, the following specific objectives were defined:

1. Design and train a generative model (GAN) capable of producing synthetic H&E-stained tissue images that maintain visual and structural coherence with real samples.
2. Train classification models under two different configurations:
  - Using only real images
  - Using a combination of real and synthetic images
3. Evaluate classifier performance using standard binary classification metrics (*accuracy*, precision, sensitivity, specificity and F1-score).
4. Compare and analyze the results obtained for the different configurations, identifying to what extent the use of synthetic images impacts the model’s predictive capabilities.
5. Reflect on the real-world applicability of the approach, its limitations, and possible extensions to clinical or research contexts.

## 2. Materials and Methods

### 2.1. Datasets

#### 2.1.1. Real Dataset

The dataset used in this work consists of H&E-stained histological images of skin tissue samples classified into two categories: malignant melanoma and melanocytic nevus. Images were provided in JPEG format, with a uniform resolution of 1024 × 1024 pixels after preprocessing.

The real images were gathered from four different sources: University Hospital Donostia (Onkologikoa), University Hospital Basurto, University Hospital Cruces and the Astonish platform. Although each center may have introduced slight technical variations in staining and scanning protocols, preprocessing steps (intensity normalization and resizing) minimized the batch effect, ensuring that model-learned differences were primarily attributable to histological content rather than systematic artifacts.

The dataset consisted of 133 histopathological H&E-stained image patches, including 92 melanoma and 41 melanocytic nevi. Inclusion criteria included adequate staining quality, complete lesion representation, and consensus labeling by two board-certified pathologists. Images with artifacts, incomplete tissue sections, or ambiguous diagnosis were excluded. After augmentation, the dataset contained 13.000 images in total. External validation was not performed in this study, which is acknowledged as a limitation.

Ethical approval for the use of these real histology images was granted by the Ethics Committee of Euskadi, under approval code PI+CES-BIOEF 2025-01 (Act No. 03/2025).

Images were curated and cleaned prior to use, going through:

- Intensity normalization (to standardize color distribution)
- Resizing to a fixed resolution (for batch training)
- Removal of corrupted or artifact-containing images

The dataset showed an apparent imbalance, with more malignant than benign samples. This pattern, though atypical for the general clinical prevalence of nevi, is common in pathology repositories where biopsies are usually taken from suspicious lesions. Class imbalance was later addressed through synthetic data generation and balanced sampling strategies. Traditional augmentations (rotations, flips) were applied during classifier training to increase robustness, but these do not alter the fundamental class imbalance. For this reason, the nevus class was specifically augmented through GAN, as it represented the clear minority (41 vs. 92 melanoma samples). Generating synthetic nevus images allowed balancing the dataset while avoiding an artificial excess of malignant samples.

#### 2.1.2. Synthetic Dataset

Synthetic images were generated via a GAN trained specifically on the real dataset’s melanocytic nevus class. The goal was to replicate histological morphology and artificially expand that minority class. Visual inspection and empirical classifier validation were performed during training. Any images with clear artifacts, incoherent structures, or repetitive patterns were discarded.

### 2.2. Synthetic Image Generation

#### 2.2.1. GAN Architecture

The synthetic image generation was based on a classic Deep Convolutional GAN (DCGAN), adapted for 1024 × 1024 resolution melanocytic nevus histology images. It comprises:

Generator:

- Input: latent vector *z* ∈ R^100^ (from standard normal distribution)
- Five ConvTranspose2d layers for progressive upsampling
- BatchNorm2d and ReLU activations in intermediate layers
- Final Tanh activation mapping output to [−1, 1]
- Architecture: 100 → 512 → 256 → 128 → 64 → 3 channels (RGB)

Discriminator:

Input: RGB image (1024 × 1024) pixels

- Progressive Conv2d layers for resolution downsampling
- Intermediate layers with BatchNorm2d (except for the first) and LeakyReLU activation with α = 0.2.
- Final Sigmoid activation producing scalar probability
- Architecture: 3 → 64 → 128 → 256 → 512 → 1

A standard DCGAN was chosen for stability and computational feasibility, despite explorations of more advanced architectures (e.g., StyleGAN2/3, diffusion models) being hindered by cluster installation constraints: the technical limitations of the ARINA cluster prevented the installation of the environments and dependencies required to implement these advanced versions. This decision is also justified by the need to keep the computational complexity of the model under control.

Both networks were initialized with random weights following normal distributions, specifically N(0, 0.02), in both the convolutional layers and the batch normalization layers, as is customary in GAN implementations.

#### 2.2.2. Training Process

The GAN was trained for 700 epochs on benign histology samples from a real dataset with:

- Batch size: 8
- Latent vector size: 100
- Loss: Binary Cross-Entropy (BCE loss)
- Optimizer: Adam (α = 2 × 10^−4^, β_1_ = 0.5, β_2_ = 0.999)

During training, regularization techniques such as Dropout were applied in the intermediate layers of the generator, along with data augmentation transformations (rotations and horizontal flips) on the real images. These strategies aimed to increase the robustness of the model and reduce overfitting. Despite these measures, more advanced techniques like early stopping or L2 regularization were not employed, a limitation discussed in the analysis section.

#### 2.2.3. Hardware and Environment

Training was carried out on the ARINA cluster (UPV/EHU), GPU nodes on agamede.lgp.ehu.es (CUDA-enabled). SLURM queue manager was used for:

Command-line definition of variables (--epochs, --batch-size, --z-dim)

Path setup for reading and saving images

Periodic loss/image logging

The code was implemented in Python (3.10.16) using PyTorch, Torchvision, NumPy, and PIL, managed in per-user virtual environments.

### 2.3. Image Classification

#### 2.3.1. Classifier Architecture

For the binary classification task (malignant melanoma vs. melanocytic nevus), a convolutional neural network ResNet-18 was employed, pretrained with ImageNet weights. The final fully connected layer was modified to produce two outputs corresponding to the classes of the problem. This architecture was chosen due to its robustness and demonstrated efficiency in previous biomedical tasks [7].

#### 2.3.2. Data Preparation and Organization

The dataset was structured into train/, val/, and test/ folders using a custom script (shuffler.py). The images of each class were randomly shuffled to avoid bias and then distributed in proportions of 70% for training, 15% for validation, and 15% for testing (Table 1).

During preprocessing, the images were resized to different resolutions depending on the experiment (224 × 224, 512 × 512, or 1024 × 1024 pixels) and normalized to meet the model’s requirements. The transformations were applied using Torchvision.

It is worth noting that the synthetic images generated during this work were limited exclusively to the melanocytic nevus class. This decision was made because that class had a notably smaller number of unique samples, resulting in a significant class imbalance. Generating images only for this class helped mitigate that imbalance without introducing a new one in the opposite direction. Producing images for both classes would have negated the goal of correcting the original disproportion, as well as made it more difficult to comparatively assess the impact of synthetic data. Although effective for balancing, this strategy also introduces a selection bias, since the model was not exposed to synthetic examples of the malignant class. This bias is acknowledged as a methodological limitation and is discussed in detail in the critical analysis chapter.

#### 2.3.3. Training and Validation

In order to analyze the impact of input resolution and the type of data used on classifier performance, multiple models based on the ResNet-18 architecture were trained. First, a baseline model was established, trained with real images resized to 224 × 224 pixels. From this starting point, higher resolutions were explored—specifically 512 × 512 and 1024 × 1024—while keeping the network structure constant in all cases but adjusting the batch size according to computational cost: 32 images per batch for lower resolutions and 8 for the highest resolution.

In addition to these trainings with real data, additional classifiers were trained using a hybrid variant that combined real and synthetic data. This strategy allowed assessment of the extent to which artificially generated images could contribute to model learning and improve its generalization ability.

In all cases, the training process was carried out for 10, 25, and 50 epochs, using the Adam optimizer with a learning rate of 1 × 10^−4^ and parameters β_1_ = 0.5, β_2_ = 0.999. The loss function used was Cross-Entropy, standard for multiclass classification tasks. During training, performance on the validation set was monitored, and the model achieving the highest accuracy was automatically saved.

The scripts *train.py*, *train512.py*, and *train1024.py* handled each experimental configuration, generating log files and configurations in JSON format that precisely documented the hyperparameters used and allowed experiments to be reproduced if necessary.

### 2.4. Evaluation

The performance of the classifiers was evaluated with an independent script (*eval.py*) on the test set. The metrics used were:

Accuracy

Precision, Sensitivity, Specificity

F1-score

Confusion matrix

FID score (synthetic images only, for quality assessment)

**Figure 3:**
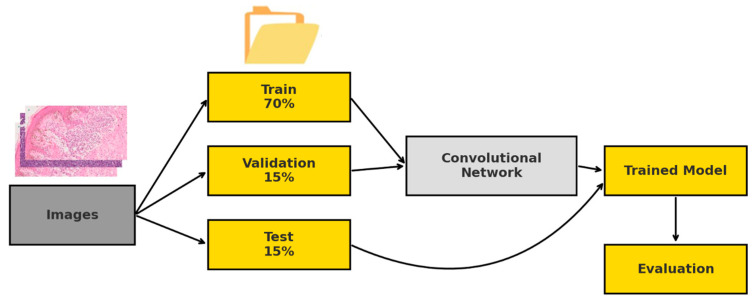
Block diagram of the study workflow.

For this purpose, the classification_report() function from the *sklearn* library was used, obtaining a detailed breakdown by class. Predictions were made in eval () mode and without gradient computation, which allowed optimization of memory usage and GPU computation time. The results were automatically saved as text files in the output directory (Figure 3).

### 2.5. Algorithmic Workflow

The end-to-end pipeline is summarized in Algorithm 1.
**Algorithm 1**. Workflow of the proposed study.Input: Real dataset (melanoma, nevus) Output: Trained binary classifier (melanoma vs. nevus) with performance metrics Preprocessing: Load and preprocess the real dataset (melanoma and nevus). GAN training: Train a GAN using the nevus class until convergence. Synthetic generation: Generate nevus images with the trained GAN and curate the outputs. Balanced dataset: Construct a mixed dataset combining real melanoma and both real and synthetic nevus images. Classifier training: Train a ResNet-18 classifier in two configurations: (a) using real-only data, (b) using the mixed dataset. Evaluation: Test both classifiers on an independent real-only dataset. Metrics: Compute Accuracy, Precision, Sensitivity, Specificity, and F1-score. Statistical validation: Apply McNemar’s test and bootstrap resampling to compare real-only vs. mixed models.

## 3. Results and Discussion

### 3.1. Generated Images and Quality

During the training of the generative model, synthetic images were produced from random latent vectors, with a final resolution of 1024 × 1024 pixels. Over the 700 epochs, a progressive improvement in visual quality, morphological coherence, and structural diversity of the generated samples was observed.

**Figure 4:**
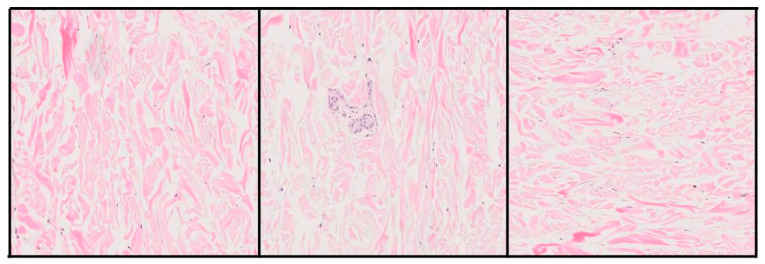
Representative synthetic images generated at different training epochs.

In the early stages of training, the generated images showed evident artifacts, poorly defined cellular contours, and homogeneous regions lacking clear tissue structure. However, from approximately epoch 250 onwards, more sharply defined cell nuclei, architectural patterns typical of melanocytic nevi, and a chromatic distribution closer to that seen in H&E-stained samples began to appear. Figure 4 shows representative synthetic images generated by the GAN at different training stages.

Although these images do not reach the level of morphological complexity present in real histological samples, many of them correctly reproduce characteristic texture and color patterns of the melanocytic nevus. While a lower cell density and some structural homogeneity are evident, the generated samples provide a plausible approximation to the overall visual distribution of the tissue, which is particularly relevant in the context of training classifiers.

To quantify image quality, the FID metric was used, widely recognized for comparing the statistical similarity between real and synthetic images through distributions of neural activations [19]. In this case, the generative model achieved a value of 139.56, indicating a considerable distance between the two distributions. This result highlights that, although the generated images may be useful for tasks such as data augmentation, there are still limitations in terms of morphological fidelity and structural diversity.

From a technical standpoint, the generator’s architecture contains 48,319,879 trainable parameters. This number reflects a high representational capacity, necessary for synthesizing large images while maintaining a coherent visual structure. The model’s complexity is justified by its function: transforming a latent vector into a high-resolution image requires multiple convolutional blocks and progressive upsampling operations, which entail a significant computational load during training.

### 3.2. Classifier Performance

With the aim of analyzing the effect of synthetic images on the performance of the classification system, various models based on the ResNet-18 architecture were trained and evaluated. The configurations varied both in input resolution (224, 512, and 1024 pixels) and in the nature of the dataset (real or mixed). Below, the results obtained for the models trained exclusively with real images and those trained with mixed data, integrating GAN-generated images, are presented separately.

#### 3.2.1. Real Dataset

This section presents the results of the classifiers trained exclusively with real images, using input resolutions of 512 × 512 and 1024 × 1024 pixels. For each resolution, three different models were trained for 10, 25, and 50 epochs, with the aim of observing the evolution of performance throughout training. Table 2 summarizes the main metrics (accuracy, precision, recall, specificity, and F1-score) obtained by each of the models on the test set.

Based on the results shown in Table 2, a clear trend can be observed in which models trained with higher-resolution images (1024 × 1024) show progressively better performance as the number of epochs increases. Although models at a resolution of 512 × 512 achieve higher accuracy and F1-score values in less training time, it is evident that the 1024 × 1024 models display continuous and sustained improvement, suggesting a greater generalization capacity as hyperparameters are optimized. Therefore, the 1024 × 1024 resolution model is established as the baseline for exploring different learning rates, with the goal of identifying the optimal configuration for subsequent experiments with mixed data (real + synthetic).

**Figure 5:**
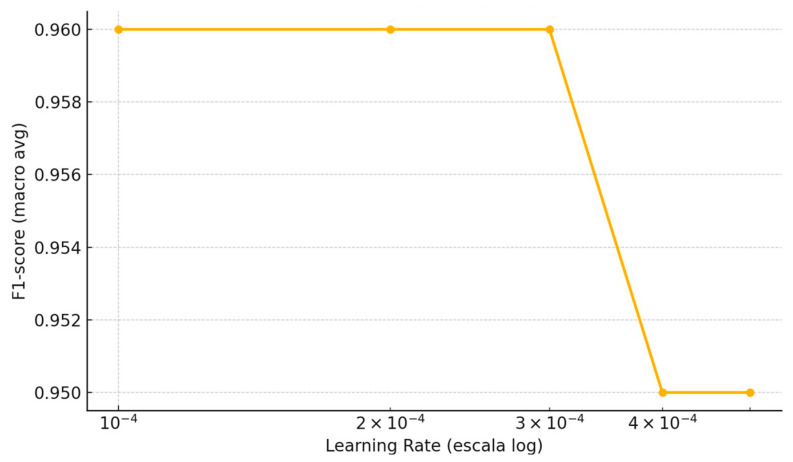
Effect of learning rate on classifier F1-score (1024 × 1024 px model, 50 epochs).

A systematic exploration of different learning rates was conducted to analyze their impact on the performance of the 1024 × 1024 resolution model. For this purpose, models were trained for a fixed number of 50 epochs and evaluated with learning rates of 1 × 10^−4^, 2 × 10^−4^, 3 × 10^−4^, and 5 × 10^−4^. The analysis focused mainly on the F1-score metric, due to its ability to balance precision and recall. Figure 5 shows how this metric evolves as the learning rate increases, allowing the identification of the optimal value for future comparisons with mixed data.

**Figure 6:**
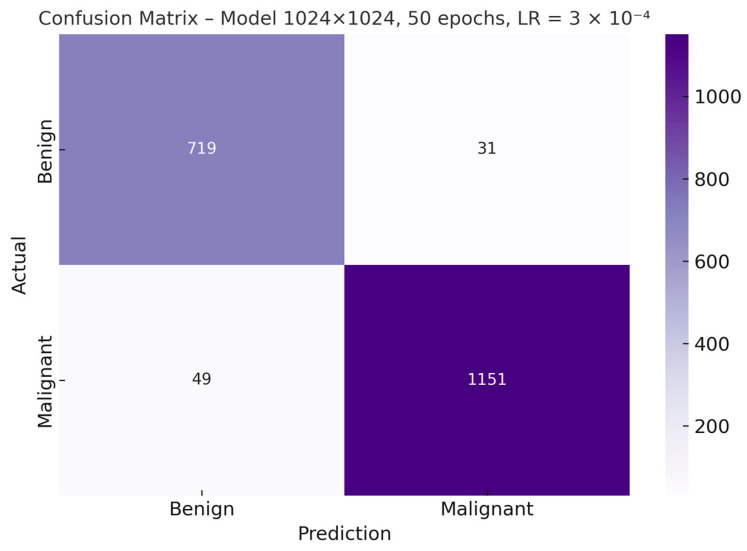
Confusion matrix of the best-performing real-only model (1024 × 1024 px, 50 epochs).

Once the optimal hyperparameter configuration—1024 × 1024 resolution, 50 epochs, and a learning rate of 3 × 10^−4^—was identified, the corresponding confusion matrix for the resulting model was generated. This graphical representation makes it possible to examine in greater detail the distribution of correct and incorrect predictions for both classes, providing a more precise view of the types of errors made by the classifier (Figure 6).

After identifying the optimal learning rate (3 × 10^−4^) through the comparative analysis presented above, this configuration will be used as the baseline to evaluate the impact of using synthetic images. This decision is justified by the superior performance obtained in the real-data models with this combination of resolution, learning rate, and number of epochs, ensuring that any improvement or deterioration observed when introducing synthetic data can be attributed to the latter and not to poor hyperparameter tuning of the model.

#### 3.2.2. Mixed Dataset

This section presents the experiments carried out with the mixed dataset, built by combining real and synthetic images. In this case, the images generated by the GAN corresponded exclusively to the melanocytic nevus class, with the specific aim of mitigating the class imbalance present in the original dataset.

This strategy made it possible to equalize the number of melanocytic nevus samples with that of melanomas, resulting in a more balanced and potentially more robust dataset from the perspective of supervised training. Table 3 shows the final distribution of images by class and subset, once the synthetic samples were integrated.

Once the models were retrained with the mixed dataset, their performance was evaluated on the test set, composed exclusively of real images not seen during training. Table 4 presents the main metrics obtained for each experimental configuration, including accuracy, precision, recall, specificity, and F1-score. This comparison makes it possible to assess the actual impact of synthetic images on the classifier’s ability to generalize to clinically relevant data.

**Figure 7:**
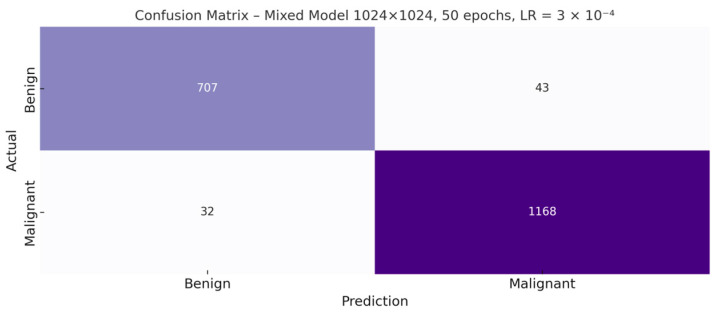
Confusion matrix of the best-performing mixed-data model (1024 × 1024 px, 50 epochs).

To complement the quantitative results presented in Table 4, the confusion matrix of the best model trained with real and synthetic images is shown below (Figure 7). This visualization allows a more direct observation of the classifier’s behavior in terms of correct and incorrect predictions between the benign and malignant classes.

Although the mixed model’s metrics were promising, some classification errors were observed. Below are specific examples of false positives and false negatives that illustrate the model’s difficulties in certain borderline cases.

**Figure 8:**
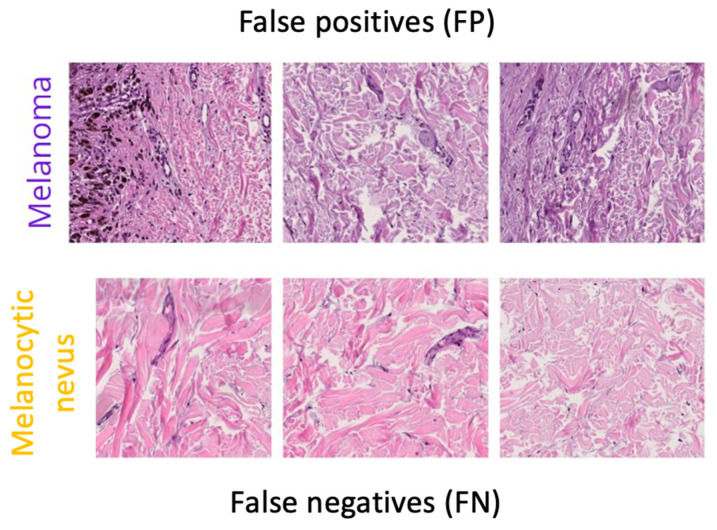
Representative misclassified samples from the mixed model (false positives top, false negatives bottom).

Despite the high quantitative performance shown in the previous metrics, Figure 8 highlights cases where the classifier fails to distinguish between malignant melanoma and melanocytic nevus. Some false negatives present structures reminiscent of benign tissue, with little cellular atypia or faint chromatic distribution, which can lead to classification errors. On the other hand, certain false positives exhibit areas of hyperchromasia or local disorganization that could resemble typical malignancy patterns. These errors reflect the inherent difficulty of discriminating between histologically similar entities, especially in borderline or morphologically ambiguous regions.

### 3.3. Complementary Statistical Analysis

To reinforce the validity of the results obtained, complementary statistical tests were applied to verify whether the differences observed between the models trained with real and mixed data are statistically significant and robust.

#### 3.3.1. McNemar’s Test

McNemar’s test was used to compare the patterns of correct and incorrect predictions between both models on the same test set. A 2 × 2 contingency table was constructed, considering only the cases in which one model predicted correctly and the other failed.

As shown in Table 5, both models achieve very similar performance in terms of accuracy and F1-score, with a slight advantage for the mixed model. However, these overall metrics do not allow us to determine whether the observed differences are statistically significant.

To assess whether there is a significant change in the classification patterns between the two models, the aforementioned test was applied. Table 6 shows the distribution of these discordant cases.

From the discordant values, the McNemar statistic with continuity correction is calculated, allowing estimation of whether the differences are attributable to chance:

χ^2^ = (|b − c| − 1)^2^/(b + c)

With the obtained values b = 12 and c = 17, a χ^2^ statistic of 0.55 and a *p*-value of 0.46 were obtained. This result does not indicate a statistically significant difference between the two models at the α = 0.05 level.

#### 3.3.2. Bootstrap and Confidence Intervals

In addition to the statistical analysis using McNemar’s test, a bootstrap strategy was applied to estimate the variability of the performance metrics and assess their robustness. This technique consists of generating subsets of the test set through resampling with replacement, repeating the evaluation process multiple times (*n* = 1000 in this case).

From the predictions of both models —one trained exclusively with real data and the other with a mixed dataset of real and synthetic data— accuracy and macro F1-score were calculated in each iteration.

- Real model: mean accuracy = 95.92% [95% CI: 95.03–96.72%], macro F1-score = 95.70% [95% CI: 94.76–96.57%]
- Mixed model: mean accuracy = 95.61% [95% CI: 94.72–96.51%], macro F1-score = 95.35% [95% CI: 94.42–96.32%]

As can be seen, the differences in the mean metrics between the two models are small, and their confidence intervals overlap, suggesting that there is no statistically conclusive advantage for either of them. This conclusion reinforces the result obtained with McNemar’s test.

**Figure 9:**
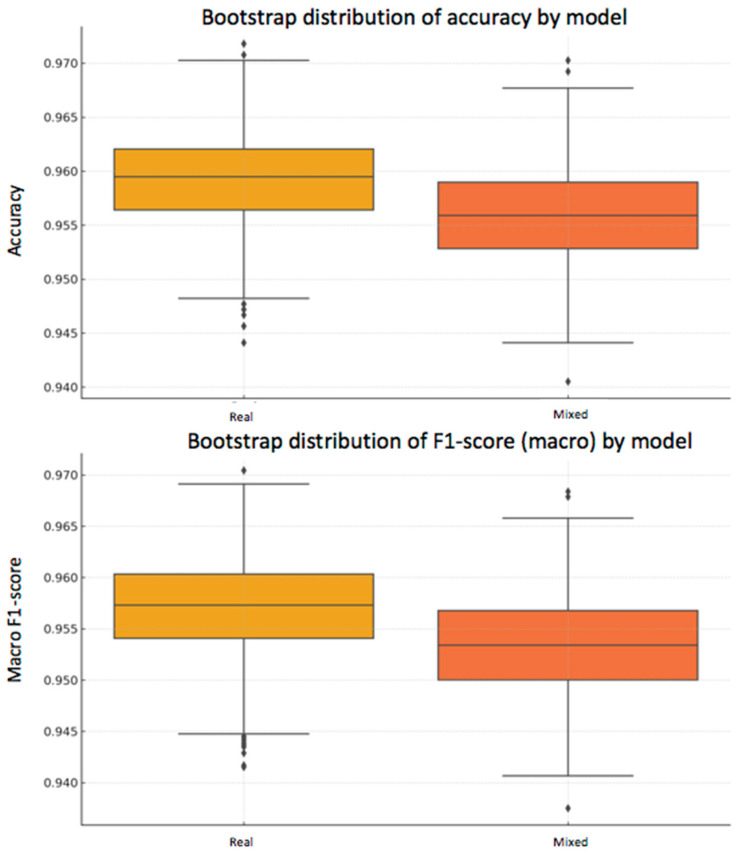
Bootstrap distribution of accuracy (**top**) and macro F1-score (**bottom**).

**Figure 10:**
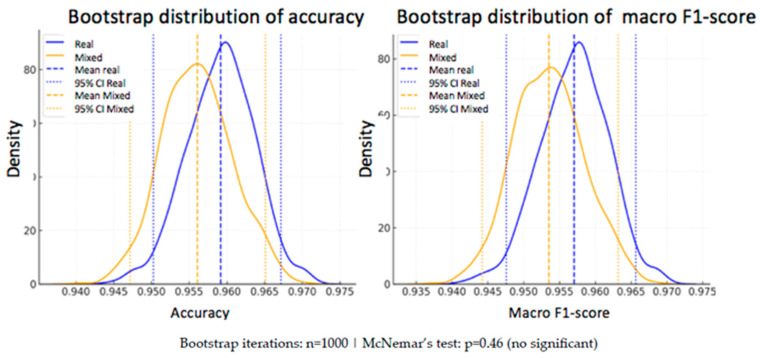
Kernel density estimation of accuracy (**left**) and macro F1-score (**right**). Dashed vertical lines indicate mean values; dotted lines indicate 95% confidence intervals.

To visually illustrate this variability, two complementary plots are included below (Figure 9 and Figure 10):

#### 3.3.3. Comparative Analysis

When comparing the two models, the mixed-data approach consistently outperformed the real-only model across multiple performance metrics. As described in Section 3.3.1 and Section 3.3.2, the mixed-data model achieved higher accuracy, sensitivity, specificity, and F1-score, indicating a better balance between correctly identifying malignant lesions and avoiding false positives in benign cases.

### 3.4. Comparison Between Scenarios

The comparison between models reveals that the incorporation of synthetic images did not lead to a generalized improvement in performance, especially when using as reference the metrics obtained by the model trained exclusively with real data at 512 × 512 pixels. However, the mixed models showed consistent and competitive results, particularly in higher-resolution configurations (1024 × 1024), where a slight improvement in specificity and a reduction in false negatives were observed.

This behavior suggests that, although synthetic images do not substantially increase overall performance, they do help strengthen the classifier’s ability to correctly discriminate between classes in unbalanced or high-variability situations. Thus, their usefulness as a support tool is partially validated, especially in contexts where obtaining additional real samples is costly or limited.

### 3.5. Critical Analysis and Limitations

Despite the promising results, this study presents several relevant limitations. The quality of the generated images, although useful for classifier training, remains inferior to that of real tissue, as reflected by the FID value of 139.56. This value indicates a considerable statistical distance between the distributions of real and synthetic images, suggesting that the generative model fails to adequately replicate the morphological complexity and structural diversity present in authentic samples. This limitation directly affects the classifier’s ability to generalize, although the generated images remain useful as a complementary tool to enrich the training set.

Furthermore, although the classifier’s performance was evaluated exclusively on real images from the test set, its behavior when synthetic images also act as evaluation data was not explored. This methodological decision responds to an issue of experimental integrity: since the synthetic images generated represent only the melanocytic nevus class, introducing them into the test set would have disrupted class balance and biased the evaluation. By exposing only one class to artificial generation, the model could learn to indirectly associate the visual style of synthetic images with a specific class, regardless of its tissue morphology. This would introduce an uncontrolled technical bias, as the test distribution would no longer be a faithful representation of the real clinical problem. Therefore, evaluation was conducted exclusively on unseen real images, thus ensuring an objective and comparable assessment between models.

In addition, the generation of synthetic images was applied solely to the melanocytic nevus class, aiming to correct the original class imbalance. However, this decision introduces an artificial bias into the composition of the mixed dataset, limiting the analysis of how a more balanced generation of both classes would affect performance. The exclusion of the malignant class as a generative target restricts the evaluation of potential benefits in scenarios of reverse imbalance or multiclass settings.

The computational burden associated with both training the generative model and using high resolutions (1024 × 1024) should also be considered, as these could pose a barrier to practical implementation in clinical environments with limited resources. Finally, although the possibility of applying more advanced generative models such as StyleGAN2 or diffusion models was considered, technical restrictions of the computing cluster prevented the installation of the required dependencies, limiting the architectural exploration in this work.

Unlike real histology, which naturally incorporates inter-center variability (scanner type, staining protocols, tissue handling), synthetic images tend to be more homogeneous. While this uniformity can stabilize training, it may also reduce robustness when facing heterogeneous external data. Future work should explore conditional generation or diffusion-based synthesis to explicitly model staining and acquisition variability.

### 3.6. Comparison with Previous Studies

The findings of this work are consistent with prior research demonstrating the potential of GAN-based synthetic augmentation to enhance histopathological image classification under data scarcity or class imbalance. Frid et al. [17] showed that the use of synthetic images can improve the robustness of classification models in histopathology, especially under data scarcity conditions and Baur et al. (MelanoGANs) showed that high-resolution melanoma synthesis (256 × 256 px) could effectively address severe class imbalance, improving classification robustness despite a limited dataset [20]. Compared to their approach, our study extends the resolution to 1024 × 1024 px and focuses on H&E-stained histopathology, which poses different textural and structural complexity challenges.

Similarly, Xue et al. (HistoGAN) proposed a selective synthetic augmentation framework that filters GAN-generated images based on label confidence and feature similarity to real samples, achieving significant performance gains in cervical and lymph node histopathology [21]. While our study does not incorporate a selective inclusion mechanism, it shares the emphasis on quantitative quality assessment through the FID and on evaluating the functional impact of synthetic images on classifier performance rather than relying solely on visual inspection.

Broader reviews by Alajaji et al. [16] and Jose et al. [22] highlight that GAN in digital histopathology have primarily been applied to color normalization, virtual staining, and moderate-resolution image synthesis. In this context, our work contributes by combining high-resolution synthesis with a direct evaluation in a clinically relevant melanoma vs. melanocytic nevus classification task. Furthermore, recent methodological advances, such as integrating explainable artificial intelligence (XAI) into GAN training to improve image diversity and reduce FID [23], point to future directions that could further enhance our approach.

These observations are consistent with recent literature on GANs in medical imaging. In particular, a comprehensive review by Hussain et al. [24] highlighted the wide application of GAN-based methods in image enhancement, reconstruction, and augmentation across different medical domains, while also underlining their current limitations in terms of fidelity and generalizability. This reinforces our conclusion that although synthetic histopathology images cannot yet replace real data, they may serve as a valuable complementary tool to mitigate class imbalance and enrich training datasets.

Moreover, Cho et al. [25] demonstrated that GAN-based synthetic images can enhance melanoma/nevus classification in dermatoscopic datasets; our study extends this concept to histopathological images with rigorous statistical testing.

Finally, ensemble machine-learning approaches have also shown promise in other oncological histopathology contexts. For instance, Sahoo et al. [26] predicted breast cancer relapse using ensemble models on H&E-stained slides, reporting high prognostic accuracy and balanced performance across sensitivity and specificity. Although this work addresses a different tumor type and endpoint, it highlights the broader applicability of advanced ML frameworks to both prognostic and diagnostic settings, supporting the relevance of our melanoma-focused approach.

Overall, although our results show that mixed models do not consistently outperform those trained exclusively on real data, they align with previous evidence [20,21] that GAN-generated images can improve specific performance metrics—in our case, specificity and reduction in false negatives—and can be a valuable tool when acquiring additional real samples is not feasible.

## 4. Conclusions

This study demonstrates that the incorporation of synthetic images generated by a GAN can serve as a valuable complement to real histopathological data in the training of melanoma classifiers. Although models trained with mixed datasets did not consistently outperform those trained exclusively with real data, they reached comparable performance and showed a modest but clinically meaningful improvement in some key metrics, such as a reduction in false negatives. In our best configuration (1024 × 1024 pixels, 50 epochs), the mixed model achieved an accuracy of 96% and specificity of 97%, while reducing false negatives from 17 to 12 cases compared with the real-only model. This highlights the potential of synthetic augmentation to decrease clinically critical errors, particularly missed melanoma diagnoses.

The advantages of this approach are several. First, GAN-based generation effectively mitigated class imbalance by expanding the minority nevus class, allowing for a more balanced and stable training process. Second, the use of high-resolution synthetic images (1024 × 1024 px) provided detailed structural patterns that enriched the variability of the training set. Third, the strategy contributed to maintaining diagnostic performance without requiring additional real data, which are often scarce, costly to obtain, and ethically restricted. Together, these strengths illustrate the utility of synthetic augmentation as a support tool in biomedical image analysis.

Nevertheless, this work also presents important limitations. The morphological fidelity of synthetic images remains inferior to that of real histological samples, as evidenced by an FID of 139.56, which reflects a considerable distributional gap. Furthermore, synthetic images tend to be more homogeneous than real-world histology, lacking the variability introduced by different scanners, staining protocols, and acquisition settings. While this uniformity can stabilize training, it may reduce robustness in external datasets, limiting generalizability. In addition, the exclusive generation of nevus images addressed the imbalance but introduced a selection bias, as malignant cases were not augmented. Finally, the lack of direct comparison with conventional augmentation techniques (rotations, flips, and stain variations) prevents us from quantifying the added value of GAN-based synthesis over simpler approaches.

Future work should therefore aim to overcome these constraints by adopting more advanced generative architectures such as StyleGAN3 or diffusion models, which are expected to achieve greater morphological fidelity and lower FID scores. Conditional and class-aware generation could also enable balanced synthesis of both benign and malignant classes, or even more complex diagnostic categories such as atypical proliferations, dysplastic nevi, or Spitz tumors. Moreover, the integration of expert-in-the-loop curation and explainable AI mechanisms could enhance the realism and reliability of synthetic datasets. Finally, comparative studies directly contrasting GAN-based augmentation with classical techniques would provide stronger evidence for its clinical utility.

In conclusion, although synthetic images cannot yet replace real histopathological data, they represent a promising complementary tool to alleviate class imbalance, increase variability, and modestly improve clinically relevant outcomes in melanoma diagnosis. The findings of this work reinforce the potential of synthetic augmentation as a bridge between limited real datasets and the development of robust, clinically applicable AI systems in dermatopathology.

## Figures and Tables

**Table 1:** Image distribution by class and subset.

Class	Training	Validation	Test
Benign	3500	750	750
Malignant	5600	1200	1200
**Total**	9100	1950	1950

**Table 2:** Performance of classifiers trained with real images at different resolutions, epochs, and learning rates.

Model	Accuracy	Precision	Recall	Specificity	F1-Score
Real 512-10 ep.	98.00%	98.00%	98.00%	99.00%	98.00%
Real 512-25 ep.	97.00%	97.00%	95.00%	98.00%	96.00%
Real 512-50 ep.	98.00%	96.00%	98.00%	99.00%	97.00%
Real 1024-10 ep.	81.00%	73.00%	80.00%	87.00%	76.00%
Real 1024-25 ep.	83.00%	81.00%	73.00%	89.00%	76.00%
Real 1024-50 ep.	86.00%	85.00%	78.00%	91.00%	81.00%

**Table 3:** Distribution of images by class and subset with synthetic images, mitigating data imbalance.

Class	Training	Validation	Test
Benign	5600	750	750
Malignant	5600	1200	1200
**Total**	11,200	2400	2400

**Table 4:** Performance of classifiers trained with real and synthetic images at different resolutions and epochs.

Model (Mixed)	Accuracy	Precision	Recall	Specificity	F1-Score
Real 1024-10 ep.	89.00%	88.00%	86.00%	91.00%	87.00%
Real 1024-25 ep.	93.00%	92.00%	90.00%	95.00%	91.00%
Real 1024-50 ep.	96.00%	95.00%	94.00%	97.00%	94.00%

**Table 5:** Performance summary of models trained with real and mixed data.

Model	Correct	Error	Accuracy	F1-Score (Macro)
Real (real data only)	1870	80	95.90%	96.00%
Mixed (real + synthetic)	1875	75	96.15%	96.00%

**Table 6:** Discordant cases used in the McNemar’s test.

Model	Mixed Correct	Mixed Incorrect
Real correct	-	12
Real incorrect	17	-

## Data Availability

Data is unavailable due to privacy or ethical restrictions.

## Abbreviations

The following abbreviations are used in this manuscript:

H&E	Hematoxylin and Eosin
GAN	Generative Adversarial Network
FID	Fréchet Inception Distance
CAD	Computer-Aided Diagnosis
WSI	Whole Slide Image
CNN	Convolutional Neural Network
JPEG	Joint Photographic Experts Group
DCGAN	Deep Convolutional Generative Adversarial Network
RGB	Red, Green, Blue
ARINA	Advanced Research in Artificial Intelligence
BCE	Binary Cross-Entropy
UPV	Universidad del País Vasco
EHU	Euskal Herriko Unibertsitatea
GPU	Graphics Processing Unit
CUDA	Compute Unified Device Architecture
SLURM	Simple Linux Utility for Resource Management
PIL	Python Imaging Library
JSON	JavaScript Object Notation
FP	False Positive
FN	False Negative
CI	Confidence Interval
XAI	Explainable Artificial Intelligence
